# Why 6-Iodouridine Cannot Be Used as a Radiosensitizer of DNA Damage? Computational and Experimental Studies

**DOI:** 10.1021/acs.jpcb.3c00548

**Published:** 2023-03-09

**Authors:** Karina Falkiewicz, Witold Kozak, Magdalena Zdrowowicz, Paulina Spisz, Lidia Chomicz-Mańka, Mieczyslaw Torchala, Janusz Rak

**Affiliations:** † Laboratory of Biological Sensitizers, Department of Physical Chemistry, Faculty of Chemistry, University of Gdańsk, Wita Stwosza 63, 80-308 Gdańsk, Poland; ‡ Laboratory of Intermolecular Interactions, Department of Bioinorganic Chemistry, Faculty of Chemistry, University of Gdańsk, Wita Stwosza 63, 80-308 Gdańsk, Poland

## Abstract

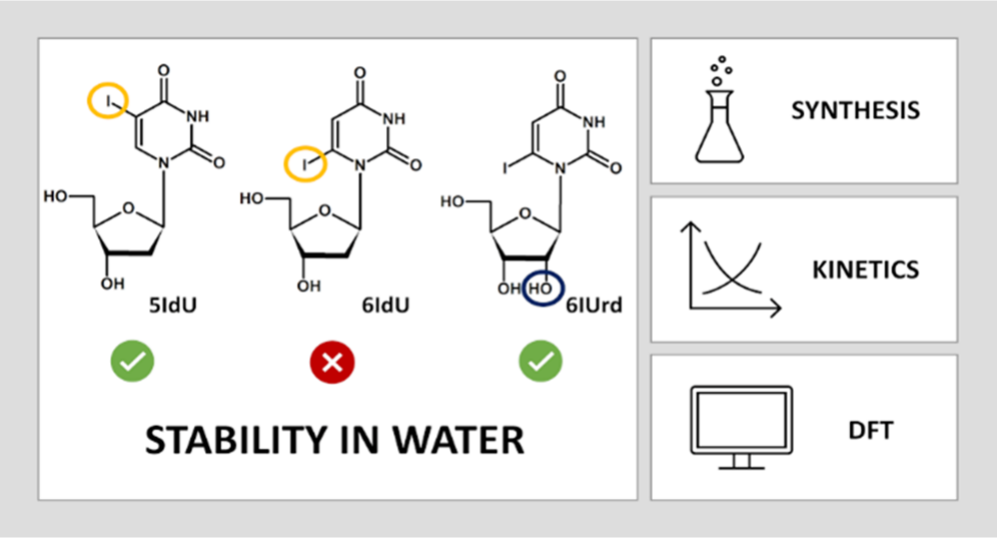

Previous density functional theory (DFT) studies on 6-brominated pyrimidine nucleosides suggest that 6-iodo-2′-deoxyuridine (6IdU) should act as a better radiosensitizer than its 5-iodosubstituted 2′-deoxyuridine analogue. In this work, we show that 6IdU is unstable in an aqueous solution. Indeed, a complete disappearance of the 6IdU signal was observed during its isolation by reversed-phase high-performance liquid chromatography (RP-HPLC). As indicated by the thermodynamic characteristics for the S_N_1-type hydrolysis of 6IdU obtained at the CAM-B3LYP/DGDZVP++ level and the polarizable continuum model (PCM) of water, 6-iodouracil (6IU) was already released quantitatively at ambient temperatures. The simulation of the hydrolysis kinetics demonstrated that a thermodynamic equilibrium was reached within seconds for the title compound. To assess the reliability of the calculations carried out, we synthesized 6-iodouridine (6IUrd), which was, unlike 6IdU, sufficiently stable in an aqueous solution at room temperature. The activation barrier for the *N*-glycosidic bond dissociation in 6IUrd was estimated experimentally using an Arrhenius plot. The stabilities in water calculated for 6IdU, 6IUrd, and 5-iodo-2′-deoxyuridine (5IdU) could be explained by the electronic and steric effects of the 2′-hydroxy group present in the ribose moiety. Our studies highlight the issue of the hydrolytic stability of potentially radiosensitizing nucleotides which, besides having favorable dissociative electron attachment (DEA) characteristics, must be stable in water to have any practical application.

## 1 Introduction

Radiotherapy involves damaging genomic DNA by exposing living cells to ionizing radiation.^1,2^ Direct effects, where ionizing photons reach the DNA molecules, are negligible for sparsely ionizing radiation, for example, using X-rays, which is commonly employed in radiotherapy.^3^ Since water is the main constituent of most cells, its radiolysis (producing mainly hydroxyl radicals and hydrated electrons) accounts for most of the secondary effects related to interactions between ionizing radiation and living matter.^4^ Hypoxic cells a hallmark of solid tumors^5^ are about 3 times more resistant to ionizing radiation than normoxic ones.^6^ To overcome the state of hypoxia which is disadvantageous from a radiotherapy standpoint, modified nucleosides (MNs) with radiosensitizing properties could be introduced into anticancer therapy.^7^ Note that some MNs can easily penetrate cell membranes, be phosphorylated in the cytoplasm, and then processed by DNA polymerases, ultimately leading to their incorporation into the newly biosynthesized DNA.^3,8,9^ Such modified DNA then becomes sensitive to hydrated electrons, one of the most abundant radiolysis products of water, which after their attachment may lead to a single-strand break (SSB).

The most thoroughly studied radiosensitizing nucleoside analogues employing the above-mentioned mechanism of DNA sensitization are 5-bromo- (5BrdU) and 5-iodo-2′-deoxyuridine (5IdU).^10−12^ The sensitizing effects of these pyrimidines are related to an efficient dissociative electron attachment (DEA) as a consequence of interactions between MNs and hydrated electrons.^13^ Indeed, hydrated electron attachment may initiate the DEA process, which will produce reactive and genotoxic nucleoside radicals. If these reactions take place inside the DNA, the reactive species resulting from DEA can lead to DNA strand breaks and finally to cell death.^14^ Recently, several new uracil derivatives and 2′-deoxyuridines with radiosensitizing properties have been proposed by our group.^15−18^

Most radiosensitizing nucleosides are 5-substituted pyrimidines (5-Pyrs).^8,9,19^ However, we have previously postulated that 6-substituted uridine or cytidine analogs could work as DNA radiosensitizers more efficiently than 5-Pyrs.^20,21^ In fact, our computational studies have demonstrated that the uridine-6-yl radical, forming with a low activation barrier DEA to the 6-substituted pyrimidine nucleosides, can abstract a hydrogen atom not only from the sugar moiety of an adjacent nucleoside in a DNA strand but also from its own 2′-deoxyribose.^20,21^ Thus, the latter process should increase the amount of strand breaks in DNA labeled with 6-substituted pyrimidines relative to DNA labeled with 5-substituted ones.

**Figure 1:**
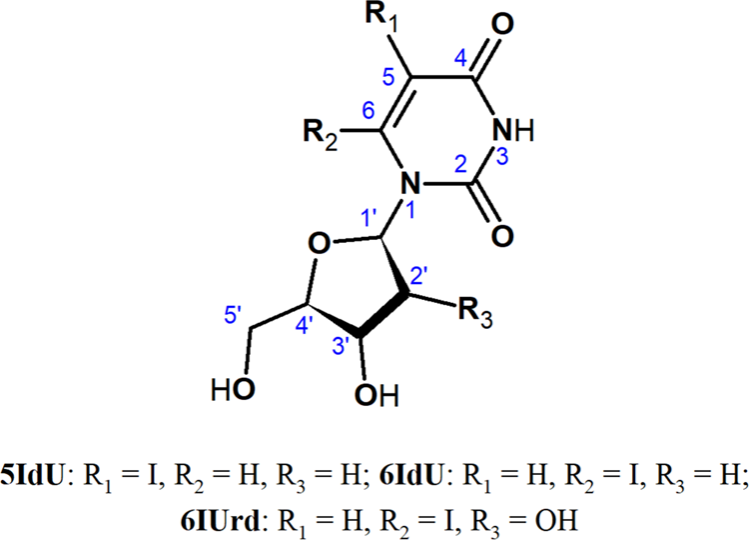
Chemical structure of 5-iodo-2′-deoxyuridine, 6-iodo-2′-deoxyuridine, and 6-iodouridine.

In this work, we have attempted to experimentally follow the concept indicated in our previous theoretical investigations,^20,21^ i.e., we have tried to synthesize 6-iodo-2′-deoxyuridine (6IdU) and carry out radiolytic studies on its aqueous solution coupled with the liquid chromatography–mass spectrometry (LC–MS) identification of radioproducts. However, it was observed that the presence of the iodine atom at the C6 position of 2′-deoxyuridine made the compound susceptible to hydrolysis already at ambient temperatures. Hence, this observation suggests that 6IdU cannot work as a radiosensitizer since any radiosensitizer has to be relatively stable in water to be transported to the cell cytoplasm and then incorporated into the cellular DNA. To explain the high observed propensity of 6IdU to hydrolysis, in addition to the attempted synthesis of 6-iodo-2′-deoxyuridine, we synthesized 6-iodouridine (6IUrd) and compared its hydrolytic behavior and that of 5-iodo-2′-deoxyuridine (5IdU) to that of 6IdU (see Figure 1 for the structures of the studied species). The mechanism of hydrolysis of the studied compounds was then described at the density functional theory (DFT) level. The thermodynamic stimuli and activation barriers were calculated for the S_N_1 substitution of the sugar moiety at the C1′ position with a water molecule. To validate the assumed level of theory, the obtained computational barrier was compared to that determined experimentally with the use of an Arrhenius plot for a relatively stable 6IUrd nucleoside. The surprising ease of hydrolysis of 6IdU compared to 6IUrd could be explained in terms of both electronic factors concerning uracil and the lack of a hydroxyl group at the C2′ position in the deoxyribose moiety.

## 2 Methods

### 2.1 Synthesis

#### 2.1.1 Synthesis of 2′,3′-*O*-Isopropylideneuridine **1** (See Figure 2)

Sulfuric acid (H_2_SO_4_, 0.65 mL) was added dropwise to a stirred suspension of uridine (1 g, 4.13 mmol) in acetone (50 mL) at room temperature (RT), and the resulting mixture was stirred for 3 h. Then, the reaction was neutralized with Et_3_N and evaporated. The raw product was purified by preparative column chromatography using CHCl_3_/MeOH 15:1 as an eluent to produce the desired compound **1** (1.05 g) in a 90.2% yield.

**Figure 2:**
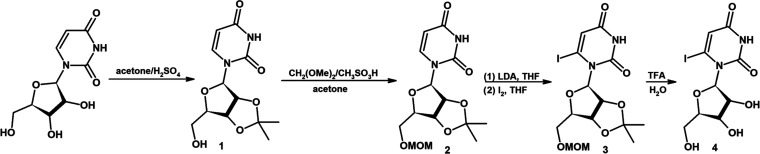
Synthesis of 6-iodouridine **4**.

#### 2.1.2 Synthesis of 2′,3′-*O*-Isopropylidene-5′-*O*-methoxymethyluridine **2** (See Figure 2)

Dimethoxymethane (13.33 mL, 150.5 mmol) and methanesulfonic acid (0.133 mL, 2.05 mmol) were added to a suspension of **1** (0.5 g, 1.76 mmol) in acetone (6.5 mL), and the resulting mixture was stirred overnight. The stirred mixture was then poured into a 25% aqueous solution of NH_4_OH and extracted with CHCl_3_. The raw product was purified by preparative column chromatography using CH_2_Cl_2_/MeOH 80:1 as an eluent to produce the desired compound **2** (385 mg) in a 66.7% yield.

#### 2.1.3 Synthesis of 6-Iodo-2′,3′-*O*-isopropylidene-5′-*O*-methoxymethyluridine **3** (See Figure 2)

A solution of **2** (300 mg, 0.91 mmol) in anhydrous tetrahydrofuran (THF, 3.5 mL) was added dropwise to a stirred solution of lithium diisopropylamide (LDA, 0.95 mL, 2.00 mmol, 2 M solution in THF) in anhydrous THF (3.7 mL) at −78 °C. After stirring for 1 h, iodine (232 mg, 0.91 mmol) in anhydrous THF (2.9 mL) was added and the mixture was stirred for an additional 5 h at −78 °C. Then, AcOH (0.45 mL) was added to quench the reaction. After bringing to RT, the raw product was extracted with AcOEt and the organic layer was washed with an aqueous solution consisting of NaHCO_3_ and brine. The product was purified by preparative column chromatography using hexane/AcOEt 7:6 as an eluent to produce the desired compound **3** (140 mg) in a 31.7% yield.

#### 2.1.4 Synthesis of 6-Iodouridine **4** (See Figure 2)

A stirred suspension of **3** (100 mg, 0.22 mmol) in water (0.77 mL) was treated with a 50% aqueous solution of trifluoroacetic acid (TFA, 1.15 mL) at 0 °C. Then, the reaction mixture was stirred for 48 h at RT. Following evaporation, the product was purified by preparative column chromatography using CHCl_3_/EtOH 10:1 as an eluent to produce the desired compound **4** (48 mg) in a 58.9% yield. Proton nuclear magnetic resonance (^1^H NMR, Bruker AVANCE III, 500 MHz, DMSO), δ: ^13^C NMR (125 MHz, DMSO), δ: 162.5, 147.6, 116.1, 115.9, 102.6, 84.9, 72.0, 69.9, 62.3 (Figure S1). High-resolution mass spectrometry (HRMS) (TripleTOF 5600+, SCIEX), *m*/*z*: [M – H]^−^ calcd for C_9_H_11_IN_2_O_6_ 368.9588, found 368.8943; ultraviolet (UV) spectrum (water), λ_max_: 270 nm.

### 2.2 Kinetic Measurements and Calculations of Kinetic Profiles

The vials with the reaction mixture (50 μL) containing 6-iodouridine in a phosphate buffer (0.1 M, pH = 7.0) were placed in an Eppendorf thermocycler for specified periods of time (see time points in Figure S8) at the following temperatures: 60, 65, 70, 75, and 80 °C. After heating, the solutions were analyzed by high-performance liquid chromatography (HPLC). The HPLC separations were performed on a Dionex UltiMate 3000 System with a diode array detector, which was set at 260 nm for monitoring the effluents. The analytes were separated on a Wakopak Handy ODS column (4.6 mm × 150 mm) using an isocratic elution with 0.1% formic acid in deionized water. The flow rate was set at 1 mL min^–1^. Each solution was injected into the HPLC instrument using an autosampler. The experiment was carried out in duplicate. The identification of the thermal analysis products was performed by mass spectrometry (LC–MS and LC–MS/MS experiments). The MS and MS/MS spectra are presented in Supporting Information Figures S2–S7. The LC–MS conditions were as follows: a TripleTOF 5600+ (SCIEX) mass spectrometer (operating in a negative mode) coupled with a Nexera X2 ultrahigh-performance liquid chromatography (UHPLC) system; a Kinetex column (Phenomenex, C18, 2.1 mm × 150 mm); a flow rate of 0.3 mL min^–1^; an isocratic elution with 0.1% formic acid in deionized water. All MS and MS/MS analyses were performed using a spray voltage of −4.5 kV and a source temperature of 300 °C.

The logarithmic values of the concentrations of 6IUrd at time zero and at different time intervals were used to establish the degradation plots. The degradation kinetic parameters (the degradation rate constants) at 60, 65, 70, 75, and 80 °C were derived from the plots. The predicted kinetics for the degradation of 6IUrd at 25 °C was extrapolated from the Arrhenius plot.

### 2.3 Quantum Chemical Calculations

The mechanisms of hydrolysis of the three considered derivatives were studied using the DFT. We applied the CAM-B3LYP functional^22^ combined with the DGDZVP++ basis set.^23^ The polarization continuum model^24^ (PCM) was used to mimic an aqueous reaction environment. All geometries were optimized and subjected to the frequency calculations for the standard state (*T* = 298 K, *p* = 1 atm). The analysis of harmonic frequencies confirmed that the stationary geometries were localized (all force constants were positive for minima; all but one were positive for the first-order transition states). The intrinsic reaction coordinate (IRC)^25^ procedure was used to verify that the transition state connected the proper minima.

The changes in Gibbs free energy (Δ*G*) for particular reactions were calculated as the difference between the Gibbs free energy of the products and substrates. The activation barriers (ΔG*) were calculated as the difference between the Gibbs free energies of the transition state and the substrate. All DFT computations were performed with the use of Gaussian 09.^26^

### 2.4 Calculations of Equilibrium Concentrations and Kinetics

The equilibrium concentrations of 5IdU, 6IdU, and 6IUrd at 25 °C were calculated using the following set of equilibrium (see eqs 1–3 and the Equilibrium Concentrations Section in the Supporting Information)
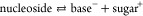
1
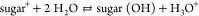
2
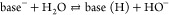
3where *K*_diss_, *K*_a_, and *K*_b_ stand for the equilibrium constants for nucleoside dissociation eq 1 and cationic eq 2 and anionic eq 3 hydrolysis, respectively.

The cationic (*K*_a_) and anionic (*K*_b_) hydrolysis constants (Table 1) were estimated employing the experimental values of acid dissociation constants (*K*_ref_) for dimethoxycarbene (MeO)_2_CHOH (p*K*_a_^27^ = −5.7) and uracil (p*K*_a_^28^ = 9.45), respectively, using eq 4.^29^
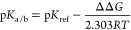
4where p*K*_a/b_ and p*K*_ref_ stand for the negative decimal logarithm of *K*_a_/*K*_b_ and *K*_ref_, respectively, while ΔΔ*G* indicates the change in Gibbs free energy for the reactions defined in Table 1.

**Table 1:** p*K* and *K* Values for the Given and Ref Compounds along with the Respective ΔΔ*G* [kcal mol^–1^]

reaction	ΔΔ*G*	p*K*	*K*
5IU^−^ + U → 5IU + U^−^	4.1	p*K*_b_ = 7.53	*K*_b_ = 3.0 × 10^–8^
6IU^−^ + U → 6IU + U^−^	7.3	p*K*_b_ = 9.91	*K*_b_ = 1.2 × 10^–10^
deoxyribose^+^ + (MeO)_2_CHOH → deoxyribose + (MeO)_2_CHOH^+^	9.1	p*K*_a_ = −12.36	*K*_a_ = 2.3 × 10^12^
ribose^+^ + (MeO)_2_CHOH → ribose + (MeO)_2_CHOH^+^	15.5	p*K*_a_ = −17.09	*K*_a_ = 1.2 × 10^17^

These equilibrium constants were, in turn, used to calculate some of the rate constants eq 5 that were required to simulate the overall kinetics of hydrolysis using eq 5
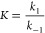
5where *k*_1_ and *k*_–1_ are the rate constants for forward and reverse reactions, respectively, while *K* is the respective equilibrium constant.

The assumed elemental reactions running in the solution of a nucleoside eqs 1–3 led to the following set of ordinary differential equations eqs 6–15
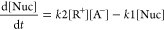
6
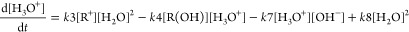
7
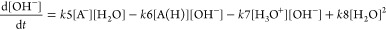
8
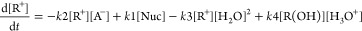
9
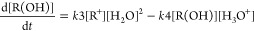
10
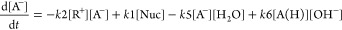
11
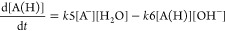
12
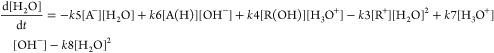
13**Additional Equations for 6IUrd Only**
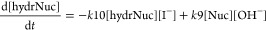
14
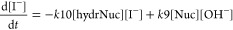
15

The abbreviations used in this system of kinetic equations were the following: Nuc—nucleoside (substrate), A(H)—nucleic base, R(OH)—sugar moiety, A^–^—anionic form of nucleic base, R^+^—cationic form of sugar moiety, and hydrNuc—6-hydroxyuridine (6OHUrd). The system of differential equations, shown in eqs 6–15, was solved with the use of the Octave program.^30,31^ The initial concentration of a nucleoside was assumed to be 10^–3^ M, and the initial concentrations of the remaining reactants were set to 0 M at time = 0. Due to the small concentrations of reactants, we assumed a constant concentration of water of 55.5 M over the course of the studied processes.^32^

## 3 Results and Discussion

**Figure 3:**
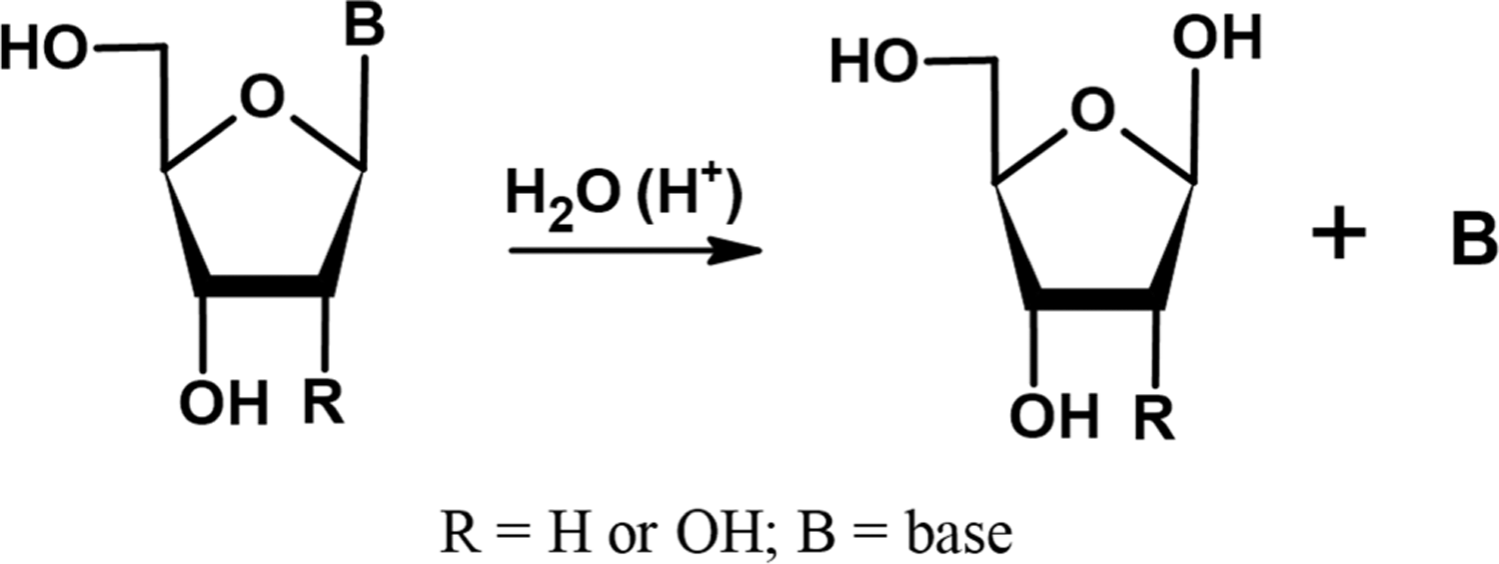
Hydrolysis of a nucleoside.^33^

One of the basic reactions that nucleosides may undergo in an aqueous solution is their hydrolysis resulting in the detachment of a nucleobase (see Figure 3).

According to Kochetkov,^33^ 2′-deoxynucleosides are hydrolyzed faster than ribonucleosides (about 100–1000 times). Indeed, the half-life of hydrolysis, τ_1/2_, for 2′-deoxyuridine (dU) amounts to 104 min, while uridine (Urd) is hydrolyzed only to a small extent even in the 3 times longer period under the same experimental conditions (5% hydrolysis in 5 h).^33^ Moreover, the identity of a nucleobase plays an important role in the stability of the *N*-glycosidic bond. Actually, the hydrolysis of purine nucleosides is faster than that of pyrimidine ones.^33^ The rate of hydrolysis is also determined by the type of substituent present in the nucleobase moiety. In fact, the degree of cleavage of the *N*-glycosidic bond for 2′-deoxycitidine (dC) is 76%, for dU—3%, and for 5XdU, where X = F, Cl, or Br, 13–17%, for a reaction occurring in 5% trichloroacetic acid for 30 min at 100 °C.^33^ Schroeder et al.^34^ demonstrated that the experimental value of the activation Gibbs free energy, ΔG*, for the heterolytic dissociation of the C1′–N1 glycosidic bond in dU amounted to 30.5 kcal mol^–1^. In turn, Przybylski et al.^35^ carried out computational studies for this reaction using the B3LYP/6-31+G(d,p) level of theory and obtained a value of ΔG* similar to that found experimentally, i.e., 28.7 kcal mol^–1^.

To explain the observed hydrolytic instability of 6IdU, we initially decided to use four functionals, including the B3LYP one, as recommended by Przybylski et al.^35^ and Chen et al.;^36^ we then calculated the free energy of hydrolysis for dU and three other derivatives considered in the current work (see Table 2). The B3LYP functional is a classical choice and has been widely used in studies of the glycosidic bond cleavage in nucleic acid-type systems.^37−39^ To describe this type of bond dissociation, Chen et al.^36^ also employed other DFT functionals, including long-range corrected hybrid functionals CAM-B3LYP^22^ and wB97XD.^40^ These authors obtained the best accuracy at the CAM-B3LYP level (the lowest mean absolute error among the considered functionals). As indicated by the results gathered in Table 2, the B3LYP and CAM-B3LYP functionals led to the most similar values compared to the experimental estimate of ΔG* for dU. On the other hand, calculations for 6IUrd proved that CAM-B3LYP provided the value that was most compatible with the experimental activation energy, as shown in the current study (see Table 2 and the Probable Mechanism of Hydrolysis Section). Therefore, we settled to use CAM-B3LYP for the ultimate estimates of the kinetic characteristics of the nucleosides considered in this study.

**Table 2:** Activation Free Energy [kcal mol^–1^] (ΔG*) of the Heterolytic Dissociation of the C1′–N1 Glycosidic Bond (Figure 3) for the Selected DFT Functionals^a^

	system			
functional	dU	5IdU	6IdU	6IUrd
B3LYP	28.2	24.3	11.9	17.9
CAM-B3LYP	**34.2**	**30.5**	**18.0**	**24.1**
ωB97XD	37.2	33.5	21.5	28.7
experimental	30.5			26.8

a All calculations were conducted using the PCM solvation model and the DGDZVP++ basis set. The CAM-B3LYP results are highlighted in bold.

### 3.1 Probable Mechanism of Hydrolysis

**Figure 4:**
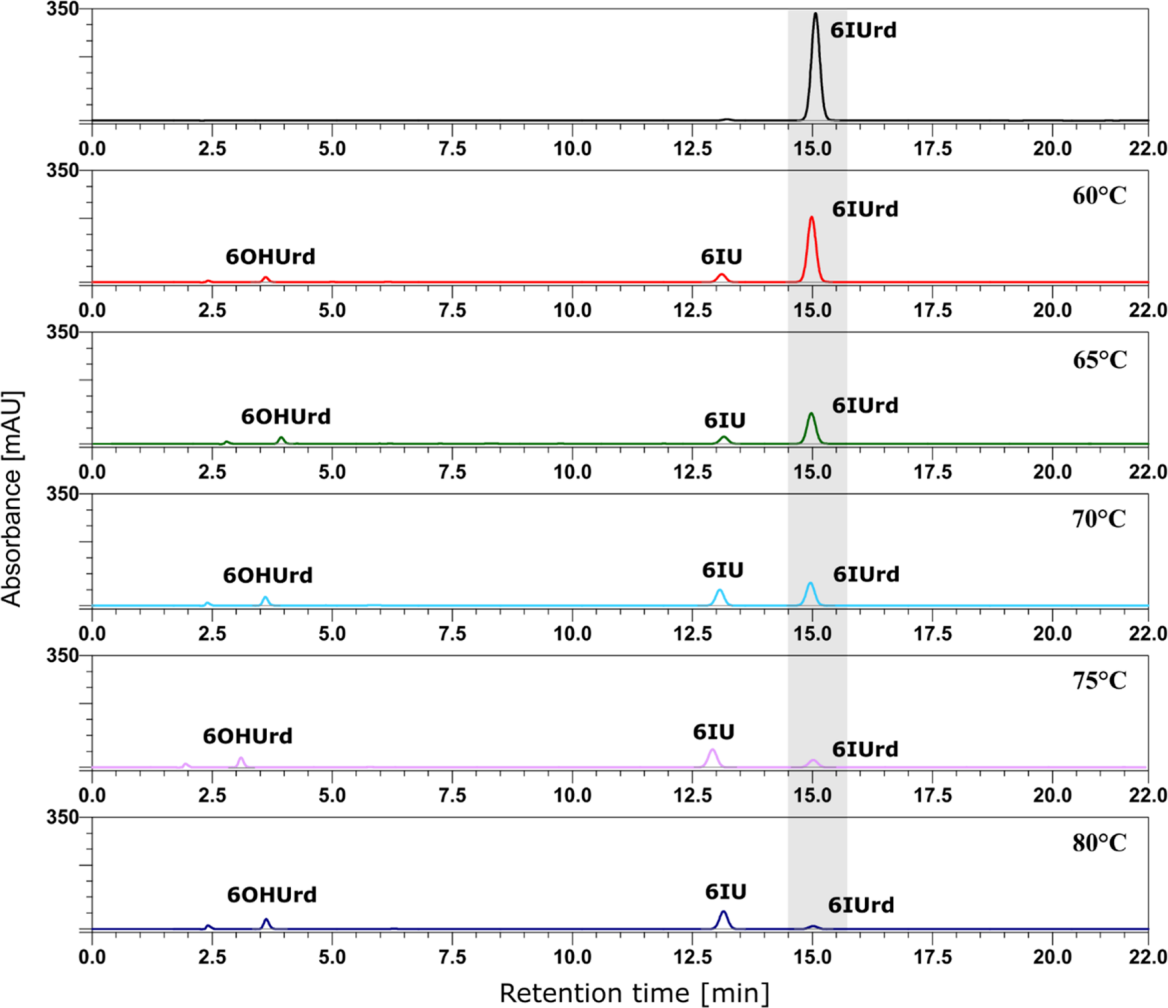
High-performance liquid chromatography (HPLC) traces of the thermal degradation of 6-iodouridine over temperatures ranging from 60 to 80 °C after 120 min of heating.

It has been reported in the literature that attempts to obtain 6-aryl-2′-deoxyuridines through a 6-iodo-2′-deoxyuridine (6IdU) derivative (both hydroxyls protected with the 1,1,3,3-tetraisopropoxydisiloxanylidene (TIPDS) group) had failed.^41^ Indeed, treating the TIPDS-protected 6IdU with tetrabutylammonium fluoride (TBAF) or ammonium fluoride (NH_4_F) produced 6-iodouracil and other degradation products.^38^ The above-mentioned findings suggest the low stability of 6IdU. Nevertheless, we attempted to synthesize 6IdU to verify whether or not we could capture the moment of its degradation. First, dU was treated with two equivalents of *t*-butyldimethylsilyl chloride (TBDMSCl) in the presence of imidazole. In the next step, the TBDMS-protected dU was introduced into a mixture of LDA and iodine at −78 °C yielding 2′,5′-di-*t*-butyldimethylsilyl-6-iodo-2′-deoxyuridine. In the last step, the deprotection of the 2′ and 5′-OH groups was performed with TBAF. The reaction was monitored by thin-layer chromatography (TLC), which showed the appearance of the desired product. We attempted to isolate this product with RP-HPLC (in a gradient of acetonitrile—ACN—and water) but registered only 6IU as a degradation product of 6IdU. Obviously, the interaction of 6IdU with water led to the swift conversion of the synthesized nucleoside into the 6-substituted base (6IU). To describe the probable mechanism of this degradation, we decided to synthesize 6IUrd which, according to the kinetic data, shown in Table 2, should have been sufficiently stable in water to allow for its kinetic stability to be assessed (ΔG* = 24.1 kcal mol^–1^ for 6IUrd vs ΔG* = 18.0 kcal mol^–1^ for 6IdU at the CAM-B3LYP level). The 6IUrd, **4**, was synthesized through a modification of procedures available in the literature^42,43^ and the protocol of its synthesis is described in detail in Section 2 (also see Figure 2). The synthesized nucleoside was then subjected to kinetic studies. The measurements were carried out at elevated temperatures so that the reaction proceeded at convenient times (a significant degradation at relatively short times). After specific periods of time had elapsed, the reaction was “frozen” by a rapid lowering of the temperature to an ambient one. Such a procedure allowed the activation barrier of hydrolysis to be determined experimentally. Figure 4 shows the HPLC traces for the reaction mixture after its 120 min conditioning at several elevated temperatures. Here, we also observed small amounts of 6-hydroxyuridine (6OHUrd; see Figure 4) in addition to the formation of 6-iodouracil. Both products were identified by LC–MS.

**Figure 5:**
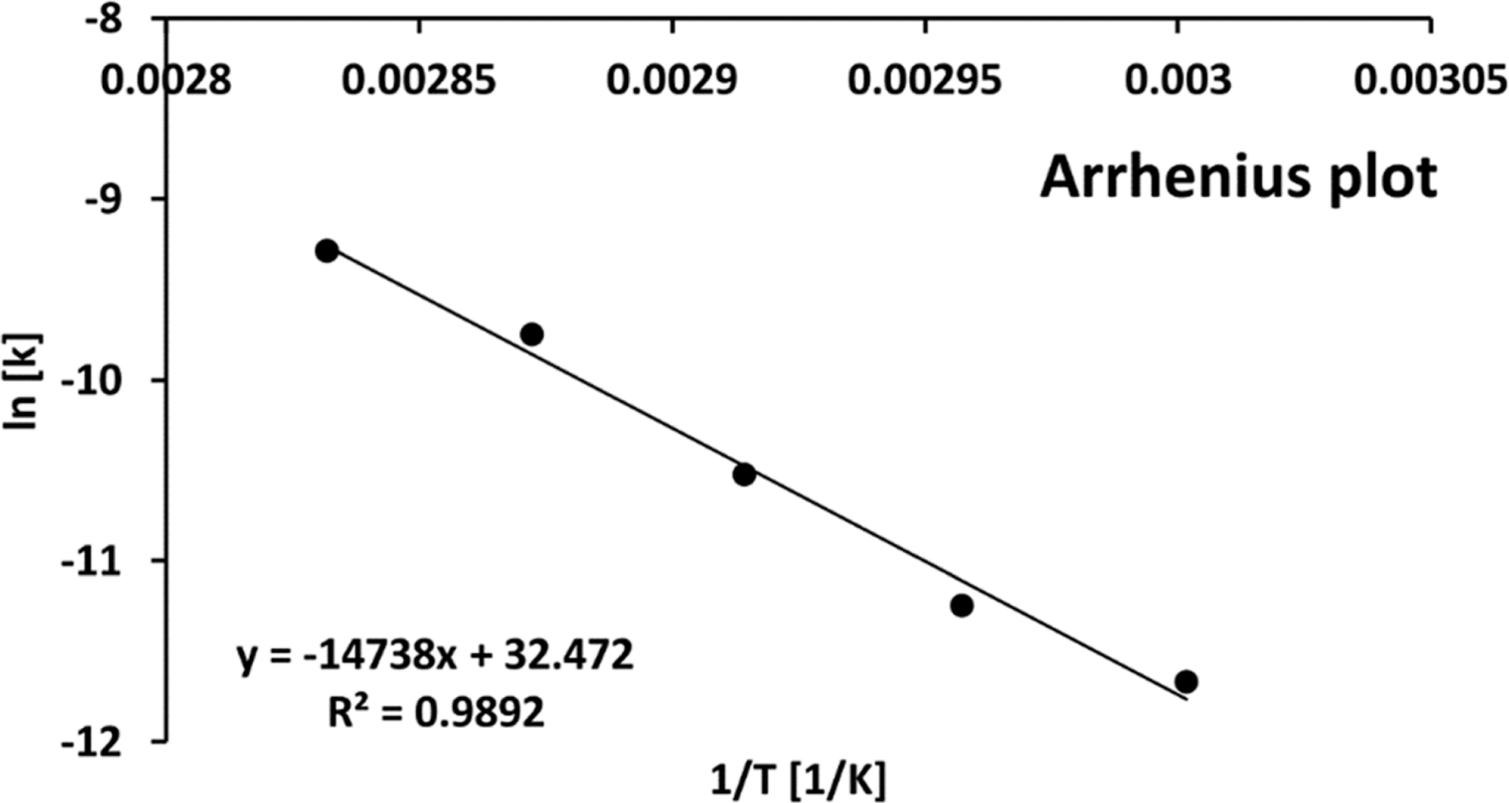
Arrhenius plot for 6IUrd hydrolysis proceeding in the 60–80 °C range.

The above-described kinetic measurements revealed a dependence of the 6IUrd concentration vs time at several temperatures, which allowed the kinetic constants for each considered temperature to be estimated (see Figure S8). In turn, these estimated rate constants allowed us to construct an Arrhenius plot (Figure 5) and, finally, to calculate the activation energy at 298 K. Derived in such a way, the experimental activation energy was equal to 26.8 kcal mol^–1^ and remained in good agreement with the ΔG* value of 24.1 kcal mol^–1^ (see Table 1) calculated at the CAM-B3LYP level.

**Figure 6:**
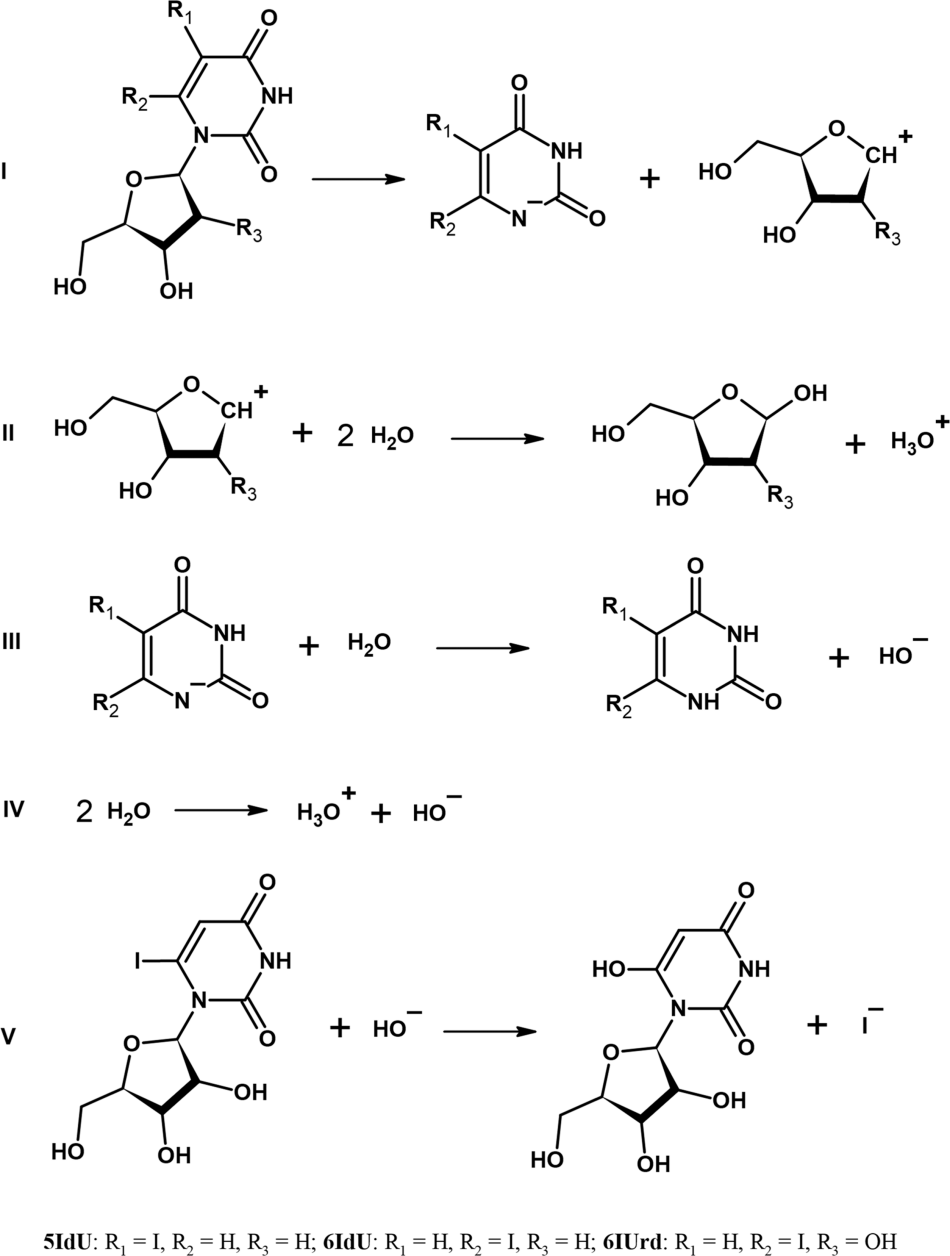
Elemental reactions for the studied mechanism of hydrolysis of the three considered derivatives.

As indicated in Figure 4, the hydrolysis of 6IUrd led to two stable products: 6IU (product of the hydrolysis of the *N*-glycosidic bond, Figure 3) and 6OHUrd. Three possible mechanisms yielding 6OHUrd were considered (Figures S9–S11). All of the analyzed reactions were thermodynamically allowed (Table 3; Δ*G* < 0).

**Table 3:** Thermodynamic and Activation Barriers [kcal mol^–1^] of 6OHUrd Formation (Figures S9–S11)

substrates	products	Δ*G*	ΔG*
6IUrd, OH^–^	6OHUrd, I^–^	–72.3	11.8
6IUrd, H_2_O	6OHUrd, HI	–11.7	43.1
6IUrd, 2H_2_O	6OHUrd, I^–^, H_3_O^+^	–10.4	39.2

However, with one water molecule, the system ended up with HI in addition to 6OHUrd, and the value of the activation barrier reached 43.1 kcal mol^–1^ (Table 3). In the model comprising two water molecules, the predicted barrier of 39.2 kcal mol^–1^ (Table 3) is somewhat lower than that calculated for a single water molecule but was still too high to allow the reaction to be completed over the experimental times (τ_1/2_ for an activation energy of the order of 40 kcal mol^–1^ at 25 °C is equal to ca. 7 × 10^9^ years). Finally, the activation barrier for the nucleophilic substitution involving the hydroxyl anion rather than water molecule(s) amounted to only 11.8 kcal mol^–1^ (Table 3). Thus, although the concentration of OH^–^ in the studied 6IUrd solution was below 10^–7^ M, the direct attack of OH^–^ on the 6IU moiety (S_N_2 mechanism) was probably responsible for the formation of 6OHUrd as a side-product.

A general mechanism for nucleoside hydrolysis which describes the hydrolysis of 6IdU, 5IdU, and 6IUrd specifically is depicted in Figure 6. The main reaction (I) consisted of a heterolytic dissociation of the C1′–N1 glycosidic bond, which led to the carbocation of the sugar moiety and carbanion of the substituted uracil. The subsequent steps involved the hydrolyses of the cationic (II) and anionic (III) products of the C1′–N dissociation. Moreover, 6-hydroxyuridine was formed through the 6IUrd hydrolysis (V).

### 3.2 Simulation of Kinetic Profiles

The equations that enabled the equilibrium concentrations in the reaction mixture to be calculated are derived in the Equilibrium Concentrations section in the Supporting Information, while the equilibrium characteristics are summarized for 5IdU, 6IdU, and 6IUrd in Table S2. However, one should note that these data do not contain any information about the time which elapsed from the dissolution of a nucleoside in water and the moment these concentrations occurred. It is worth emphasizing that these times were measures of the stability of the studied compounds and would help decide about the possible usage of a nucleoside as a radiosensitizer. Hence, to determine the time profiles of hydrolysis, we performed kinetic simulations by integrating a system of ordinary differential eqs 6–15 and using rate constants obtained as indicated in Section 2.4 for each of the three studied nucleosides (see Table S1).

**Figure 7:**
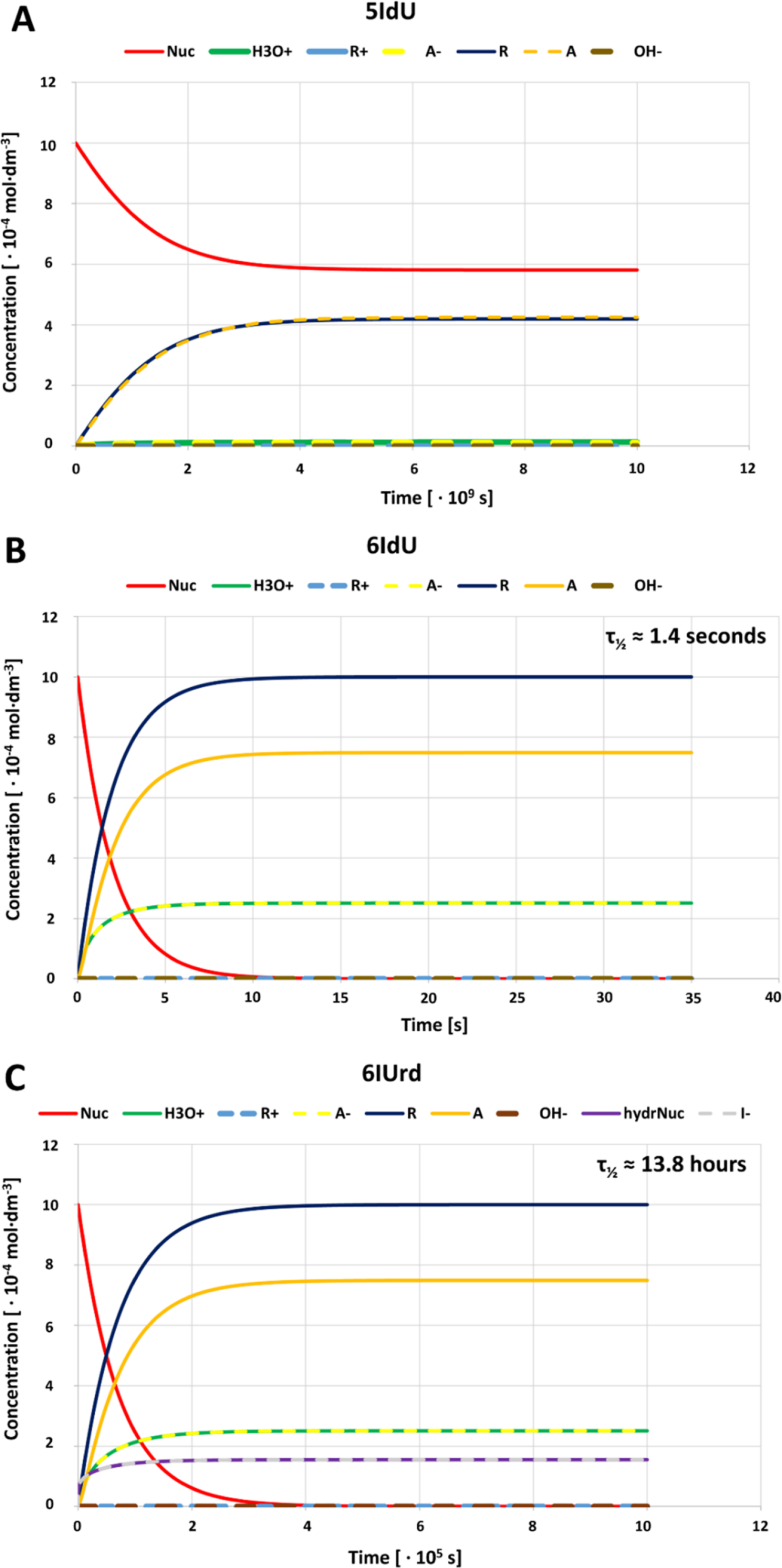
Concentration–time plots for 5IdU (A), 6IdU (B), and 6IUrd (C). Nuc—nucleoside, A—base, R—sugar moiety, A^–^ and R^+^—anionic and cationic forms of the base and sugar moieties.

Figure 7 shows the concentration profiles plotted against the time of hydrolysis reaction. For all studied systems, a decrease in the concentration of a nucleoside (Nuc) and a proportional increase in the concentration of a base (A) and sugar product (R) were observed.

The equilibrium concentration of 6IU assumed a significantly lower value than the initial concentration of 6IUrd as was also indicated by the experimental data (see Figure 4). In the case of 5IdU, the equilibrium was attained before the complete hydrolysis of the substrate. Our kinetic simulations led to τ_1/2_ being equal to 13.8 h and 1.4 s for 6IUrd and 6IdU, respectively, while for 5IdU, τ_1/2_ was not defined. One could, however, determine the time after which the equilibrium concentrations were reached (42% of the substrate decomposes) to be ca. 126 years. The data obtained thus explain the observed stabilities of 6IUrd and, in particular 5IdU, and the significant instability of 6IdU in an aqueous solution. To confirm the accuracy of the kinetic simulations performed here, the calculated equilibrium concentrations shown in Table 4 were compared with those obtained through the kinetic calculations. As demonstrated by these data, both concentrations were in excellent agreement.

**Table 4:** Equilibrium Concentrations [mol dm^–3^] (Equilibrium; for Details, See the Equilibrium Concentration section in the Supporting Information) and the Corresponding Values Resulting from the Integration of Eqs 6–15 (Kinetics)

	5IdU		6IdU		6IUrd	
reagent	equilibrium	kinetics^a^	equilibrium	kinetics^a^	equilibrium	kinetics^a^
nucleoside [Nuc]	5.8 × 10^–4^	5.8 × 10^–4^	3.6 × 10^–10^	9.0 × 10^–10^	2.4 × 10^–10^	4.1 × 10^–9^
base [A]	4.1 × 10^–4^	4.3 × 10^–4^	7.5 × 10^–4^	7.5 × 10^–4^	7.5 × 10^–4^	7.5 × 10^–4^
base anion [A^–^]	1.2 × 10^–5^	1.1 × 10^–5^	2.5 × 10^–4^	2.5 × 10^–4^	2.5 × 10^–4^	2.5 × 10^–4^
sugar moiety [R]	4.2 × 10^–4^	4.2 × 10^–4^	1.0 × 10^–3^	1.0 × 10^–3^	1.0 × 10^–3^	1.0 × 10^–3^
sugar moiety cation [R^+^]	2.2 × 10^–21^	2.3 × 10^–21^	1.1 × 10^–19^	1.1 × 10^–19^	2.1 × 10^–24^	2.1 × 10^–24^
[OH^−^]	8.5 × 10^–10^	7.8 × 10^–10^	4.0 × 10^–11^	4.0 × 10^–11^	4.0 × 10^–11^	4.0 × 10^–11^
[H_3_O^+^]	1.2 × 10^–5^	1.3 × 10^–5^	2.5 × 10^–4^	2.5 × 10^–4^	2.5 × 10^–4^	2.5 × 10^–4^
6OHUrd [hydrNuc]						1.6 × 10^–4^
iodide anion [I^−^]						1.6 × 10^–4^

a Small differences between the equilibrium and kinetic concentrations of some regents were due to the finite integration time of the kinetic equations.

At first glance, such a profound influence of subtle structural differences between the studied systems seems surprising, but it could be explained by steric and electronic effects. Indeed, since the hydration energies were almost identical for the studied nucleosides, the lower stability of 6IdU relative to 5IdU was associated with the larger steric hindrance in the 6-substituted derivative (the repulsion between the iodine atoms and the sugar residue was much smaller in 5IdU); in turn, the lower stability of the ribose cation relative to the 2′-deoxyribose one made the half-life of 6IdU significantly shorter than that of 6IUrd. Indeed, the free energy of nucleoside dissociation (eq I in Figure 6) was ca. 6.4 kcal mol^−1^ lower at the CAM-B3LYP/PCM level for 6IdU than for 6IUrd. The presence of the OH group in the 2′ position of the ribose moiety, exerting a negative inductive effect, was probably responsible for the lower stability of their respective carbocations.

## 4 Summary

This paper presents a combination of theoretical and experimental studies devoted to understanding the aqueous stabilities of specific modified nucleosides with potentially radiosensitizing properties. This investigation was prompted by our previous findings that suggested that 6-substituted uracils were better radiosensitizers than their 5-substituted analogues.

Our attempts to synthesize 6IdU were unsuccessful, which was ascribed to the low stability of the compound in water (the synthesized nucleoside was isolated and purified with RP-HPLC using an ACN:water mobile phase).

The experimental activation energy and as a consequence, the half-life and the calculated free activation energy remained in good agreement for 6IUrd. The half-life of the latter determined by kinetic simulations amounted to 13.8 h (see Figure 7). On the other hand, predicted τ_1/2_’s of 1.4 s and 126 years were estimated for 6IdU and 5IdU, respectively. The above-mentioned differences in τ_1/2_ were likely due to dissimilarities between steric and electronic effects of the considered molecules.

Since slight structural differences between nucleosides led to huge variations in their stabilities in water, to design a working radiosensitizer, one has to estimate the aqueous stability of considered nucleosides alongside performing calculations of their DEA profiles^3^ before the actual synthesis. Therefore, the proposed kinetic model should be employed in future studies devoted to the rational engineering of new radiosensitizers.

## Supporting Information Available

^1^H NMR spectrum; high-resolution mass spectra of 6-iodouridine, 6-hydroxyuridine, and 6-iodouracil; reaction profiles for 6OHUrd formation from 6IUrd and OH^–^, single water molecule and two water molecules; calculations for equilibrium concentrations and table with their values; and system of differential equations for the kinetic profiles in the Octave program (PDF)
