# Supplementary material for: Why 6-Iodouridine Cannot Be Used as a Radiosensitizer of DNA Damage? Computational and Experimental Studies
Source: J Phys Chem B. 2023 Mar 9;127(11):2565–74. doi: 10.1021/acs.jpcb.3c00548 (PMC10041638; doi:10.1021/acs.jpcb.3c00548)
Supplement: Supplementary file 1 — jp3c00548_si_001.pdf [file jp3c00548_si_001.pdf]

Supporting Information

Why 6-Iodouridine Cannot Be Used as a Radiosensitizer of DNA Damage? Computational and Experimental Studies

Karina Falkiewicz,[1] Witold Kozak,[1] Magdalena Zdrowowicz,[1] Paulina Spisz,[1,2] Lidia Chomicz-Mańka,[1] Mieczyslaw Torchala,[1] Janusz Rak[1*]

[1]Laboratory of Biological Sensitizers, Department of Physical Chemistry, Faculty of Chemistry, University of Gdańsk, Wita Stwosza 63, 80-308 Gdańsk, Poland

[2]Laboratory of Intermolecular Interactions, Department of Bioinorganic Chemistry, Faculty of Chemistry, University of Gdańsk, Wita Stwosza 63, 80-308 Gdańsk, Poland

[*]To whom correspondence should be addressed, e-mail: janusz.rak@ug.edu.pl

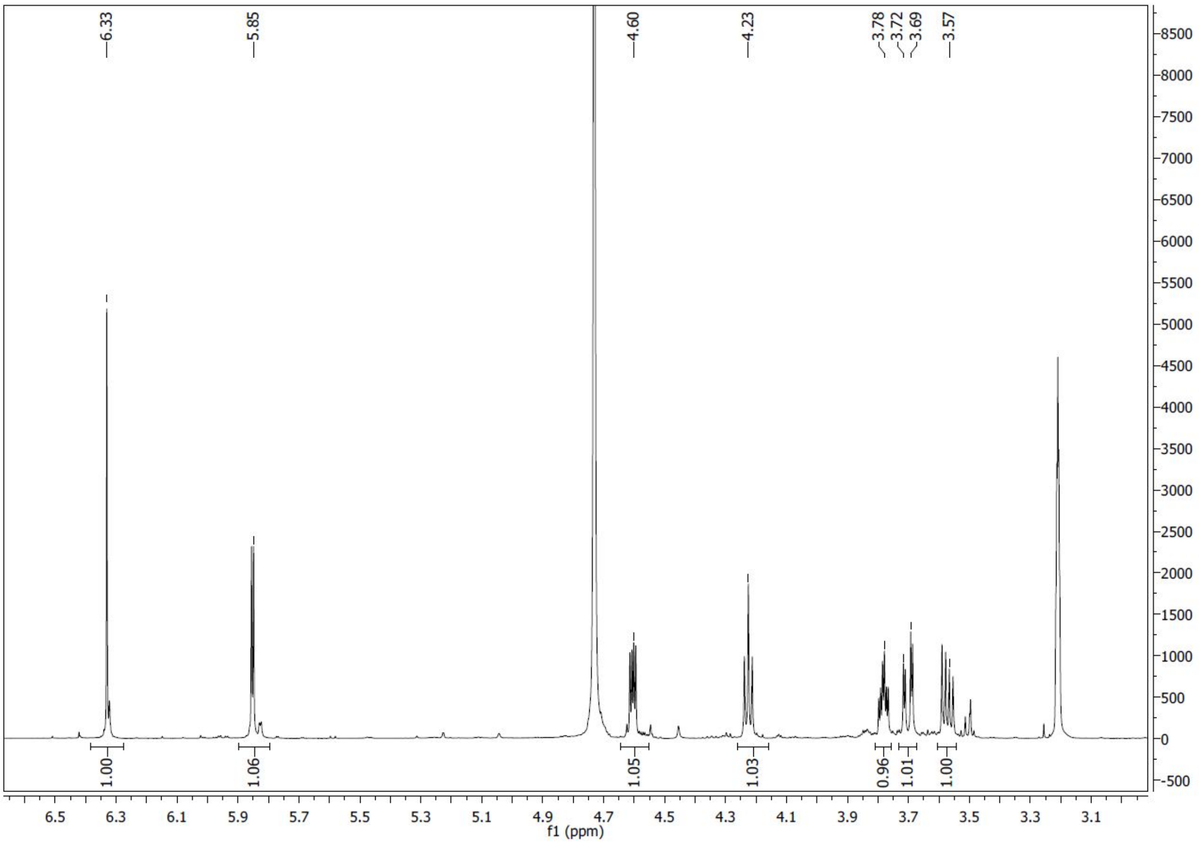

Figure S1. ^{1}H NMR spectrum of 6-iodouridine.

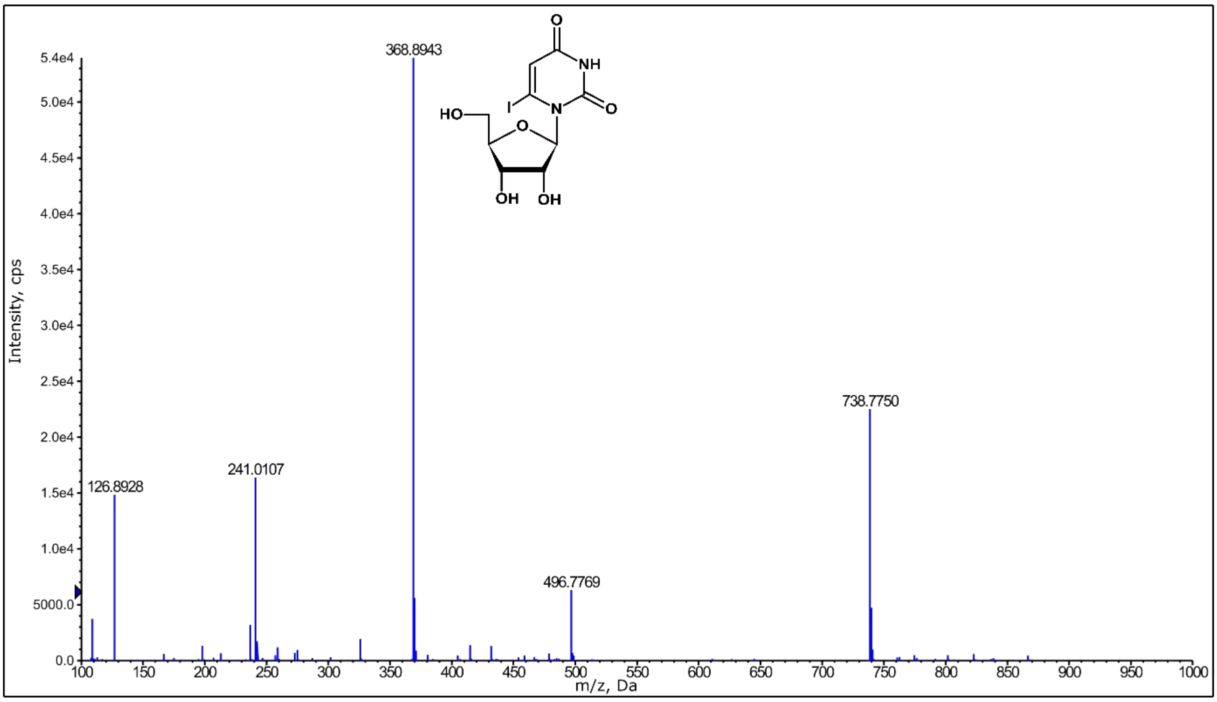

Figure S2. MS spectrum of 6-iodouridine.

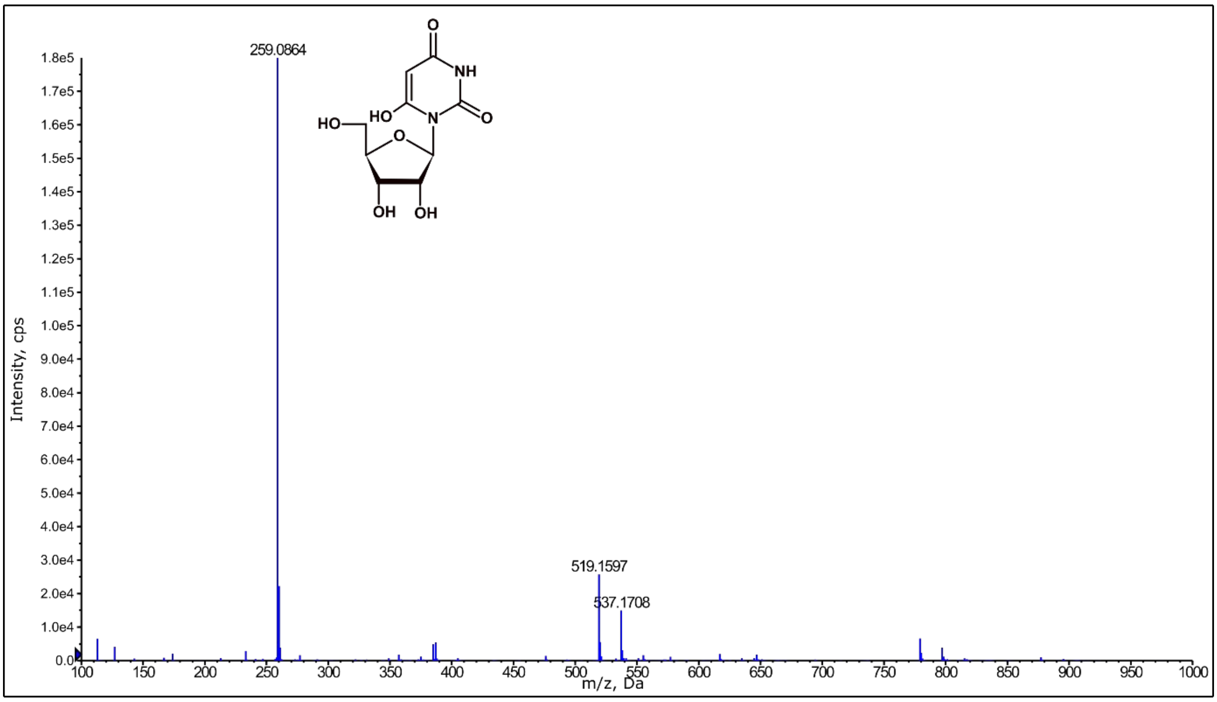

Figure S3. MS spectrum of 6-hydroxyuridine.

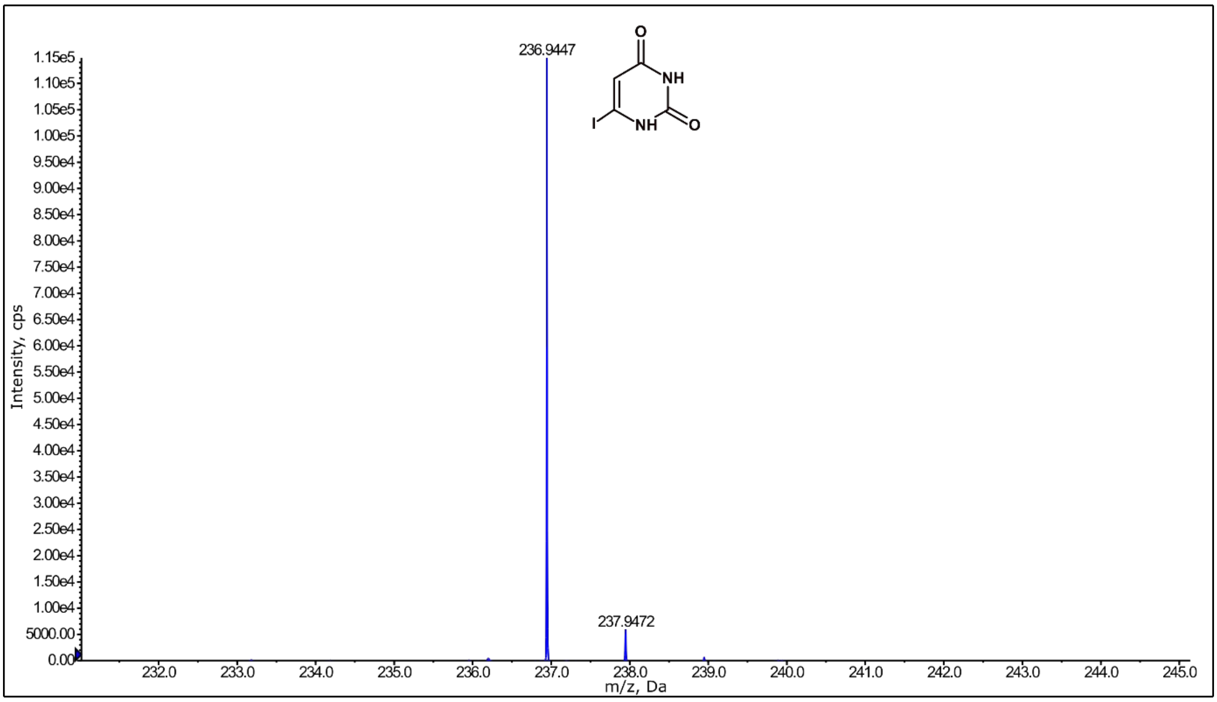

Figure S4. MS spectrum of 6-iodouracil.

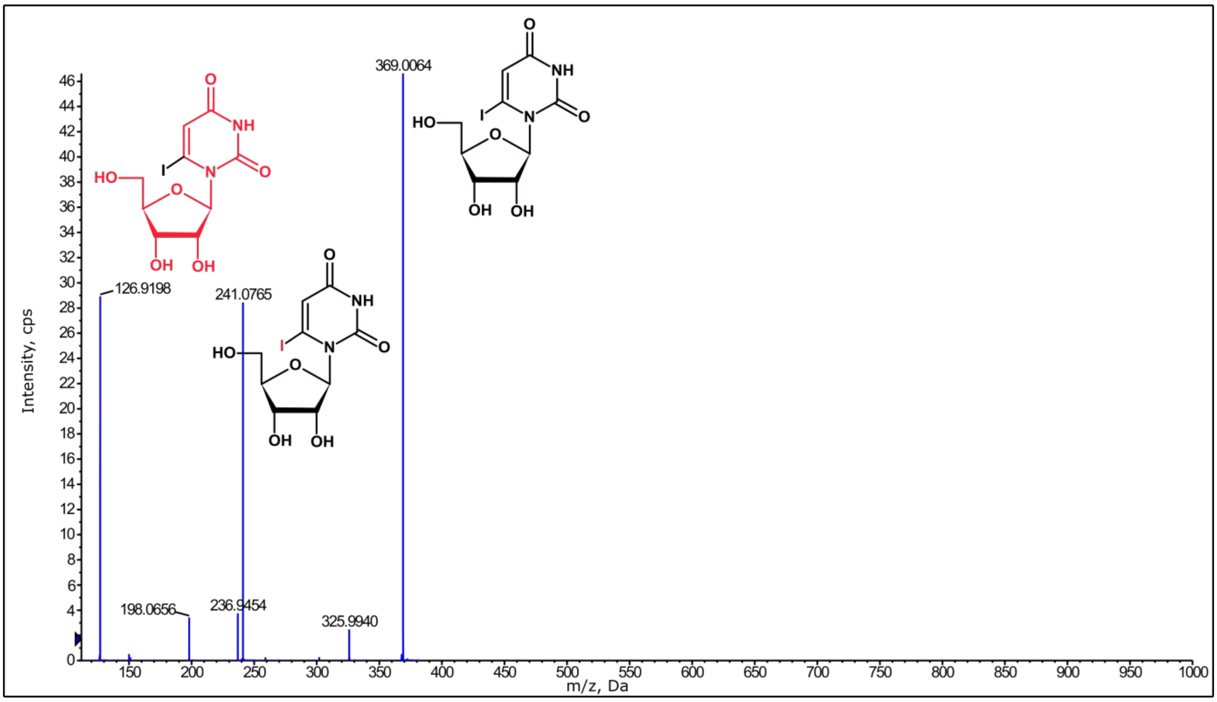

Figure S5. MS/MS spectrum of 6-iodouridine with ion identities.

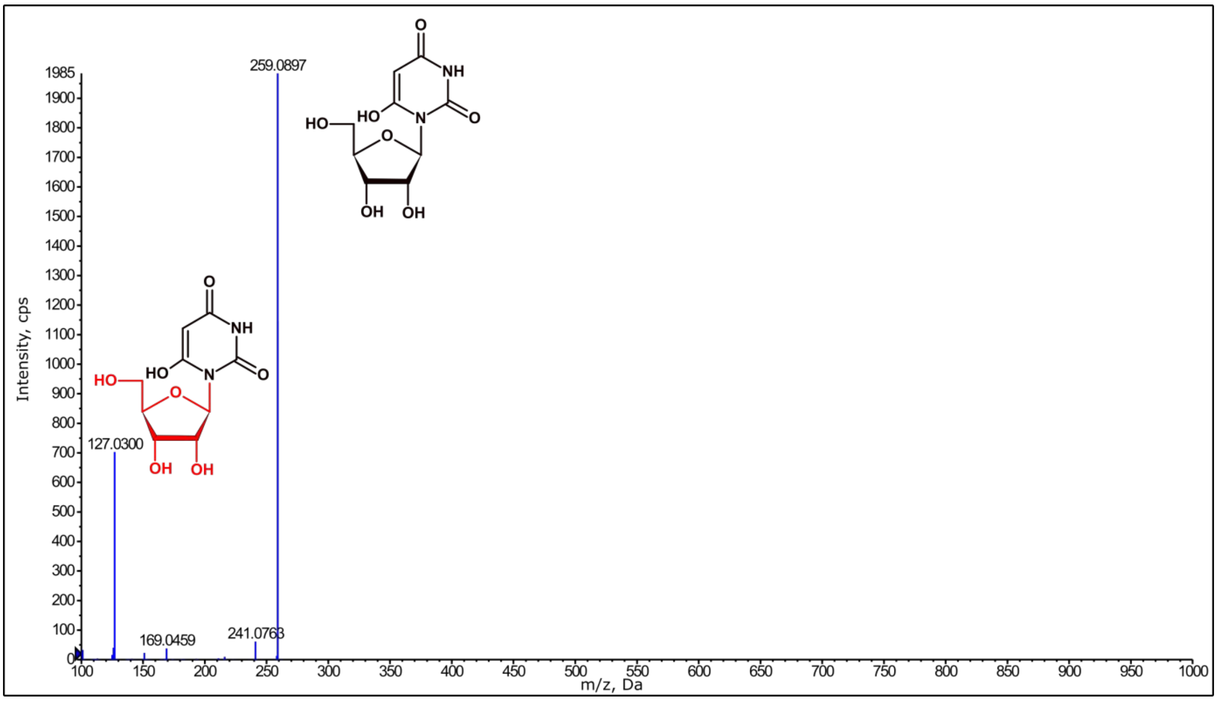

Figure S6. MS/MS spectrum of 6-hydroxyuridine with ion identities.

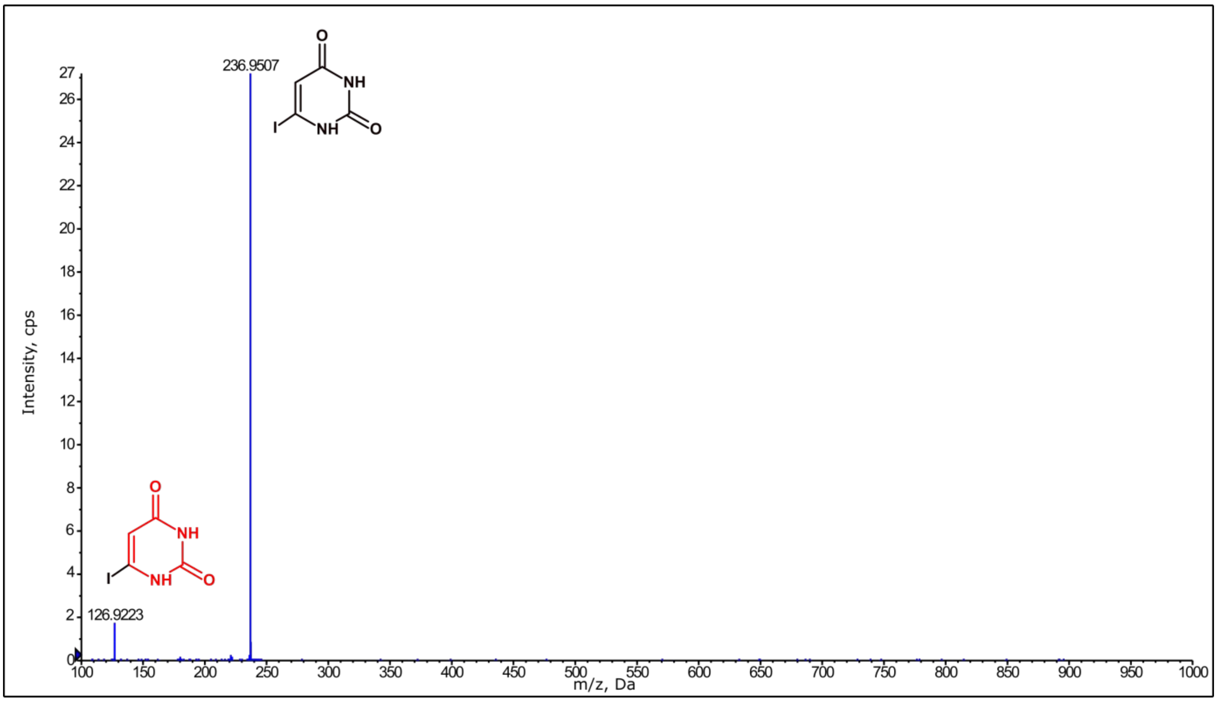

Figure S7. MS/MS spectrum of 6-iodouracil with ion identities.

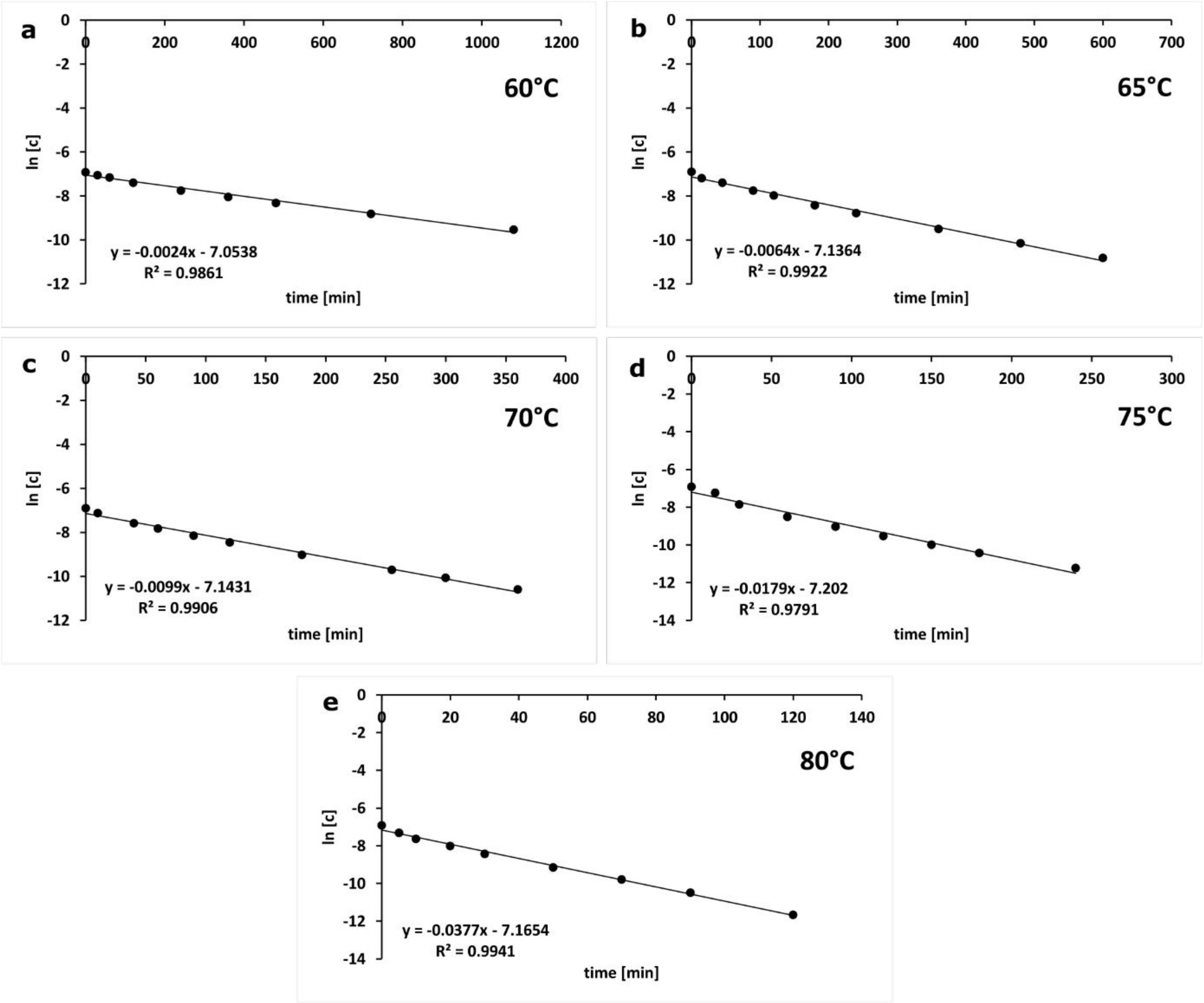

Figure S8. Logarithm of 6IUrd concentration versus time of hydrolysis at several chosen temperatures.

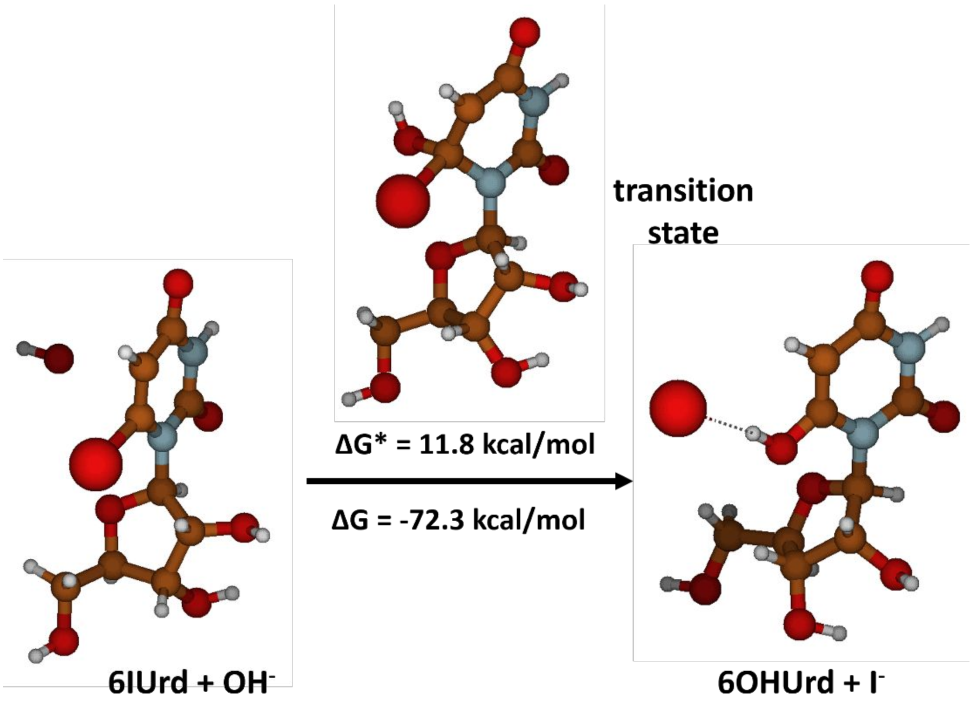

Figure S9. Thermodynamic (ΔG) and kinetic (ΔG*) barriers of 6OHUrd formation from 6IUrd and OH^-.

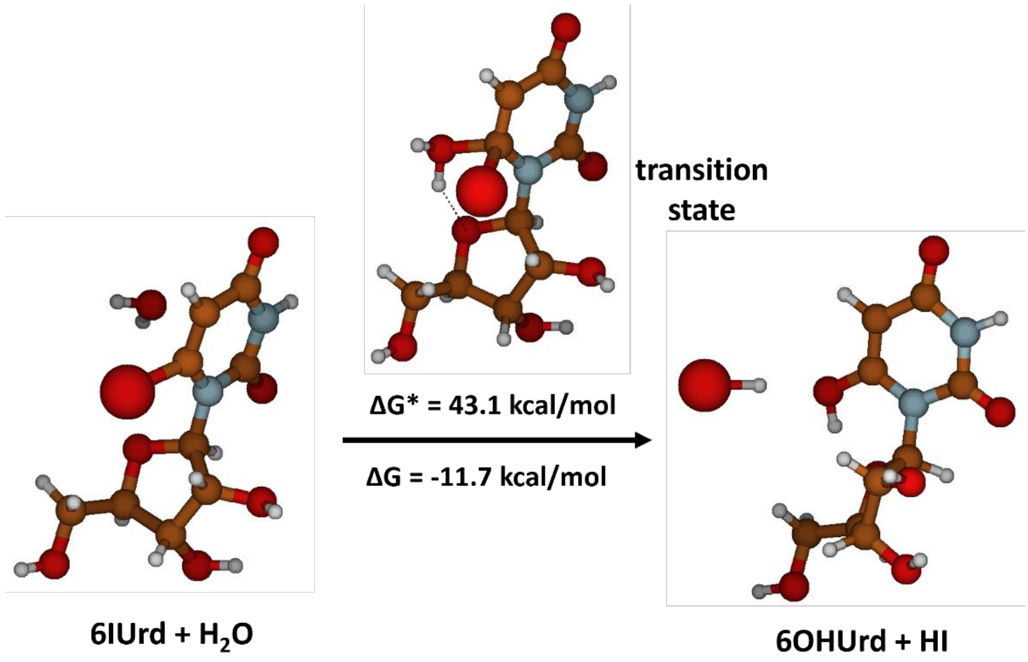

Figure S10. Thermodynamic (ΔG) and kinetic (ΔG*) barriers of 6OHUrd formation from 6IUrd and single water molecule.

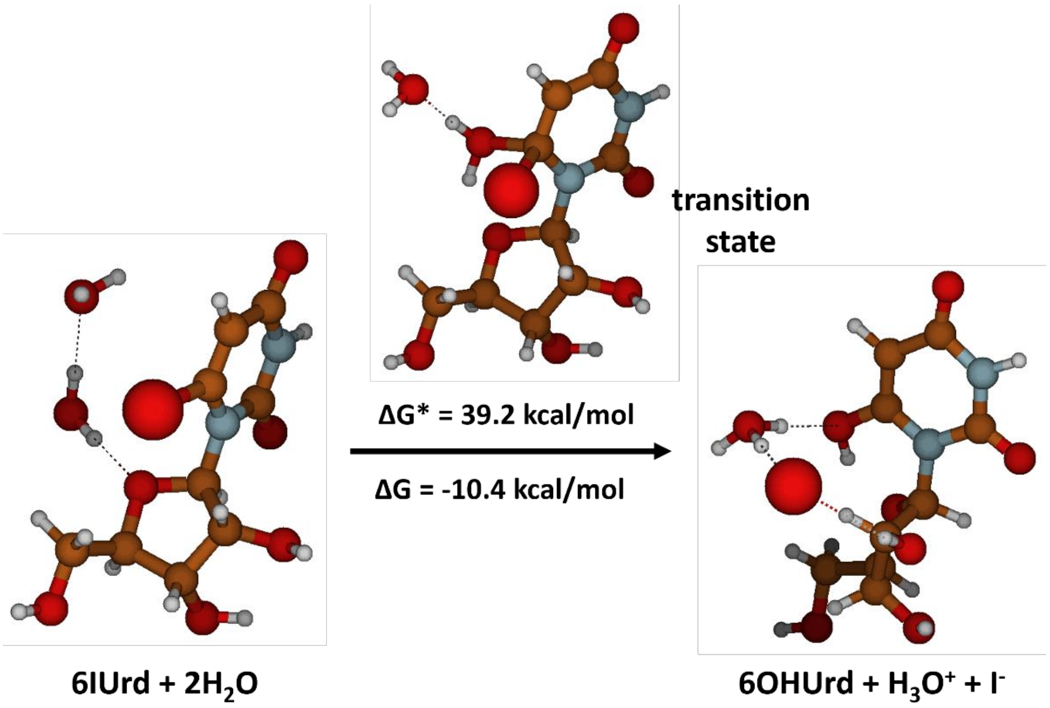

Figure S11. Thermodynamic (ΔG) and kinetic (ΔG*) barriers of 6OHUrd formation from 6IUrd and two water molecules.

Table S1. Rate constants (T = 298.15 K) employed in the kinetic model (Equations (6)-(15)) for elementary reactions of hydrolysis (Figure 7).

Compound	Reaction	Equilibrium constant*	Rate constants**
5IdU	I	$K_{diss} = 4.4 \cdot 10^{-23}$	$k_1 = 2.9 \cdot 10^{-10}$<br>$k_2 = 6.6 \cdot 10^{12}$
	II	$K_a = 7.5 \cdot 10^8$	$k_3 = 1.0 \cdot 10^{10}$ [S2]<br>$k_4 = 1.3 \cdot 10^1$
	III	$K_b = 5.4 \cdot 10^{-10}$	$k_5 = 5.0 \cdot 10^9$ [S2]<br>$k_6 = 9.3 \cdot 10^{18}$
6IdU	I	$K_{diss} = 7.6 \cdot 10^{-14}$	$k_1 = 5.0 \cdot 10^{-1}$<br>$k_2 = 6.6 \cdot 10^{12}$
	II	$K_a = 7.5 \cdot 10^8$	$k_3 = 1.0 \cdot 10^{10}$ [S2]<br>$k_4 = 1.3 \cdot 10^1$
	III	$K_b = 2.2 \cdot 10^{-12}$	$k_5 = 5.0 \cdot 10^9$ [S2]<br>$k_6 = 2.3 \cdot 10^{21}$
6IUrd	I	$K_{diss} = 2.2 \cdot 10^{-18}$	$k_1 = 1.4 \cdot 10^{-5}$<br>$k_2 = 6.6 \cdot 10^{12}$
	II	$K_a = 4.0 \cdot 10^{13}$	$k_3 = 1.0 \cdot 10^{10}$ [S2]<br>$k_4 = 2.6 \cdot 10^{-4}$
	III	$K_b = 2.2 \cdot 10^{-12}$	$k_5 = 5.0 \cdot 10^9$ [S2]<br>$k_6 = 2.3 \cdot 10^{21}$
	V	$K = 1.0 \cdot 10^{53}$	$k_9 = 1.5 \cdot 10^4$<br>$k_{10} = 1.5 \cdot 10^{-49}$
	IV	$K_{H2O} = 3.3 \cdot 10^{-18}$	$k_7 = 1.4 \cdot 10^{11}$ [S2]<br>$k_8 = 4.6 \cdot 10^{-7}$

* Equilibrium constants K_a and K_b include concentration of water equal to 55.5 mol·dm^{-3} [S1].
** Unit: [s^{-1}] or [dm^3 · (mol · s)$^{-1}$] depending on the order of the reaction.

Rate constant k_7 for autodissociation of water (**Equation 13**) was assumed according to known literature values[S1,S2] and to be equal to $k_7 = 1.4 \cdot 10^{11}$, for the reaction $OH^- + H_3O^+ \rightarrow 2H_2O$. Concentration of water $[H_2O]$ = 55.5 mol·dm^{-3}, $[OH^-][H_3O^+] = 10^{-14}$ mol·dm^{-3}. The water dissociation constant) can be calculated as in Equation S1:

$$K_{H_2O} = \frac{[OH^-][H_3O^+]}{[H_2O]^2} = \frac{10^{-14}}{55.5^2} \approx 3.3 \cdot 10^{-18} \quad \text{(S1)}$$

The constant k_8 (see Equation S4) can be calculated from the relationship presented at Equations S2-S3:

$$k_7[H_3O^+][OH^-] = k8[H_2O]^2 \quad \text{(S2)}$$

$$\frac{[H_3O^+][OH^-]}{[H_2O]^2} = \frac{k_8}{k_7} = K_{H_2O} \quad \text{(S3)}$$

$$k_8 = k_7 \cdot K_{H_2O} = 1.4 \cdot 10^{11} \cdot 3.3 \cdot 10^{-18} \approx 4.6 \cdot 10^{-7} \quad \text{(S4)}$$

Equilibrium concentrations

The equilibrium concentrations of individual species in aqueous solutions of 5IdU, 6IdU and 6IUrd were calculated using the following equations (S5-S16), consistent with the proposed mechanism of hydrolysis:

$$C_{acid} = [Sugar^+] + [Sugar(OH)] \quad (S5)$$

$$C_{base} = [Base^-] + [Base(H)] \quad (S6)$$

where Sugar(OH) and Base(H) are the sugar moiety and the nucleobase, while $Sugar^+$ and $Base^-$ are their cationic and anionic forms, respectively. It was assumed, that those concentrations are equal, thus (Equations S7, S8):

$$C_{acid} = C_{base} = C \quad (S7)$$

$$[OH^-] + [Base^-] = [Sugar^+] + [H_3O^+] \quad (S8)$$

Equilibrium constants can be represented as follows (Equation S9):

$$K_a = \frac{[Sugar(OH)][H_3O^+]}{[Sugar^+]} \quad K_b = \frac{[Base(H)][OH^-]}{[Base^-]} \quad K_{diss} = \frac{[Sugar^+][Base^-]}{[Nucleoside]} \quad K_w = [H_3O^+][OH^-] \quad (S9)$$

Further derivations lead to further transformations (Equation S10):

$$[Sugar^+] = \frac{C_{acid}[H_3O^+]}{K_a + [H_3O^+]}, \quad [Base^-] = \frac{C_{base}[OH^-]}{K_b + [OH^-]} \quad (S10)$$

The concentration will be the difference between the initial concentration and the molar concentration of the nucleoside (Equation S11):

$$C = C_o - [Nucleoside] = C_o - \frac{C_{acid}[H_3O^+]C_{base}[OH^-]}{K_{diss}(K_a + [H_3O^+])(K_b + [OH^-])} = C_o - \frac{C_{acid}C_{base}K_w}{K_{diss}(K_a + [H_3O^+])(K_b + [OH^-])} = C_o - \frac{C^2K_w}{K_{dys}(K_a + [H_3O^+])(K_b + [OH^-])} \quad (S11)$$

After transformation, the form of a quadratic equation can be obtained (Equation S12):

$$\frac{C^2K_w}{K_{diss}(K_a + [H_3O^+])(K_b + [OH^-])} + C - C_o = 0 \quad (S12)$$

Equation can be solved by calculating the delta (Equation S13) and one of its (positive) solutions (Equation S14):

$$\Delta = 1 + 4C_o \frac{K_w}{K_{diss}(K_a + [H_3O^+])(K_b + [OH^-])} \tag{S13}$$

$$x_1 = \frac{-1 + \sqrt{\Delta}}{2 * \frac{K_w}{K_{diss}(K_a + [H_3O^+])(K_b + [OH^-])}} = \frac{(-1 + \sqrt{\Delta})K_{diss}(K_a + [H_3O^+])(K_b + [OH^-])}{2K_w} \tag{S14}$$

Assuming that solution x_1 (Equation S14) is equal to equation (S7) and based on equations (S8) and (S10), this leads to equation (S15):

$$[H_3O^+] + \frac{x_1[H_3O^+]}{K_a + [H_3O^+]} = [OH^-] + \frac{x_1[OH^-]}{K_b + [OH^-]} \tag{S15}$$

and after substituting the solution x_1:

$$[H_3O^+] + \frac{[H_3O^+](-1 + \sqrt{\Delta})K_{diss}(K_a + [H_3O^+])(K_b + [OH^-])}{2K_w(K_a + [H_3O^+])} = [OH^-] + \frac{[OH^-](-1 + \sqrt{\Delta})K_{diss}(K_a + [H_3O^+])(K_b + [OH^-])}{2K_w(K_b + [OH^-])}$$

(S16)

Knowing that $[OH^-] = \frac{K_w}{[H_3O^+]}$:

$$[H_3O^+] + \frac{[H_3O^+]^2(-1 + \sqrt{\Delta})K_{dys}\left(K_b + \frac{K_w}{[H_3O^+]}\right) - 2K_w^2 - (-1 + \sqrt{\Delta})K_{diss}(K_a + [H_3O^+])K_w}{2K_w[H_3O^+]} = 0$$

(S17)

The above equation was solved using the Octave program[S3], and the equilibrium concentration of H_3O^+ ions allowed to determine the equilibrium concentrations of the remaining reagents (**Table S2**).

Table S2. Equilibrium concentrations [mol·dm^{-3}] (equilibrium) of individual reagents participating in the hydrolysis of iodouridines.

Reagent	5IdU	6IdU	6IUrd
nucleoside [Nuc]	$5.8 \cdot 10^{-4}$	$3.6 \cdot 10^{-10}$	$2.4 \cdot 10^{-10}$
base [A]	$4.1 \cdot 10^{-4}$	$7.5 \cdot 10^{-4}$	$7.5 \cdot 10^{-4}$
base ion [A$^-$]	$1.2 \cdot 10^{-5}$	$2.5 \cdot 10^{-4}$	$2.5 \cdot 10^{-4}$
sugar moiety [R]	$4.2 \cdot 10^{-4}$	$1.0 \cdot 10^{-3}$	$1.0 \cdot 10^{-3}$
sugar moiety ion [R$^+$]	$2.2 \cdot 10^{-21}$	$1.1 \cdot 10^{-19}$	$2.1 \cdot 10^{-24}$
[OH$^-$]	$8.5 \cdot 10^{-10}$	$4.0 \cdot 10^{-11}$	$4.0 \cdot 10^{-11}$

$[H_3O^+]$	$1.2 \cdot 10^{-5}$	$2.5 \cdot 10^{-4}$	$2.5 \cdot 10^{-4}$
6OHUrd [hydrNuc]	-	-	-
iodide $[I^-]$	-	-	-

References

[S1] Jencks, W.P., Caplow M., Gilchrist M., Kallen R.G. Equilibrium Constants for the Synthesis of Hydroxamic Acids. *Biochemistry* **1963**, *2*, 1313-1320.

[S2] Pigoń, K., Ruziewicz, Z. Chemia Fizyczna. t. 1, Warszawa: PWN, **2005**, 478-479.

[S3] Eaton, J.W., Bateman, D., Hauberg, S., Wehbring, R. GNU Octave version 5.2.0 manual: a high-level interactive language for numerical computations. **2019**.
